# In Vitro Evaluation of Novel Synthetic Benzimidazolium–Chalcone Hybrids: Antioxidant and Regenerative Effects in Diabetic Wound Healing

**DOI:** 10.1007/s12010-026-05712-y

**Published:** 2026-05-08

**Authors:** Burcu Demirbağ, Hakan Ünver, Ayça Kara, Adem Necip, Metin Yıldırım

**Affiliations:** 1 Health Services Vocational School, Medical Laboratory Techniques Program, Mersin University, Mersin, Türkiye Turkey; 2 Department of Chemistry, Faculty of Science, Eskisehir Technical University, Eskisehir, Türkiye; 3 Genome and Stem Cell Research Center, Erciyes University, Kayseri, Türkiye Turkey; 4 Department of Pharmacy Services, Vocational School of Health Services, Harran University, Sanliurfa, Türkiye; 5 Department of Biochemistry, Faculty of Pharmacy, Çukurova University, Adana, Türkiye Turkey

**Keywords:** Chalcone, Wound healing, HDF-1, Diabetes, α-glucosidase

## Abstract

The prevalence of diabetes mellitus (DM) is increasing daily worldwide. DM patients suffer from numerous complications, including the development of chronic wounds that can lead to amputation. These complications necessitate innovative approaches. As part of these innovative approaches, this study synthesized four chalcone derivatives and evaluated their activities in an in vitro diabetic wound model. Several spectroscopic techniques, including 1 H and 13 C NMR and HR-MS, were used to confirm the structures of the newly synthesized compounds. After investigating the inhibition of α-glucosidase in HDF-1 cells treated with D-glucose (50 mM), MTT tests and scratch tests were performed. Tumor necrosis factor-alpha (TNF-α), interleukin-1 beta (IL-1β), total antioxidant status (TAS), and procollagen type-1 were analyzed using an ELISA kit. Additionally, proliferation (Ki67), inflammatory (Nuclear factor kappa B; NFκB), and growth factor (Platelet-derived growth factor subunit A; PDGFA) markers in fibroblasts were assessed by immunohistochemistry. All compounds exhibited strong α-glucosidase inhibitory activity, with IC₅₀ values ranging from 1.115 to 1.612 µg/mL. Cytotoxicity analysis demonstrated that all compounds were biocompatible, maintaining over 85% cell viability in HDF-1 cells. Under diabetic conditions, treatment significantly improved cell viability and promoted wound closure, particularly in C4. In addition, the compounds reduced pro-inflammatory cytokines TNF-α and IL-1β while increasing TAS and procollagen type I levels. Immunocytochemical findings revealed enhanced Ki67 and PDGFA expression and decreased NFκB activation in treated groups. Molecular docking analysis supported the experimental findings by demonstrating favorable binding interactions with α-glucosidase. In conclusion, chalcone derivatives—particularly compound C4—promote diabetic wound healing through multitarget mechanisms involving anti-inflammatory, antioxidant, and pro-regenerative effects, highlighting their strong therapeutic potential.

**Supplementary Information:**

The online version contains supplementary material available at 10.1007/s12010-026-05712-y.

## Introduction

Diabetes is a metabolic disease characterized by persistently high blood sugar levels. Diabetic wounds, one of the serious complications associated with diabetes, are characterized by chronic inflammation, decreased angiogenesis, dysfunctional fibroblast activity, and impaired tissue repair [1]. In diabetes, hyperglycemia-induced dysfunction of Glucose Transporters (GLUTs) can impair energy-dependent repair functions by reducing glucose uptake in keratinocytes, fibroblasts, and immune cells [2, 3]. High glucose suppresses fibroblast migration and proliferation, impairs tissue repair by reducing collagen synthesis and growth factors, and increases oxidative stress and chronic inflammation [4, 5]. Consequently, wound failure and even foot or lower extremity amputation can occur [6]. Impaired wound healing presents complex challenges that necessitate new therapeutic strategies targeting the interconnected phases of inflammation, proliferation, angiogenesis, and remodeling [6, 7]. These phases are regulated by interactions among keratinocytes, fibroblasts, endothelial cells, inflammatory cells, and a network of bioactive mediators, including extracellular matrix components, growth factors, and cytokines [8, 9]. However, because this process is impaired in diabetic patients, a comprehensive understanding of the precise impact of wounds on individuals with diabetes is crucial to optimizing wound healing outcomes by facilitating the implementation of personalized interventions [10].

Diabetes pathophysiology involves not only hyperglycemia but also oxidative stress, inflammation, and altered cellular signaling. Recent research highlights natural bioactive compounds with multi-targeted effects for diabetes management. For instance, methanolic extracts of *Trigonella foenum-graecum* seeds lower blood glucose and enhance antioxidant defenses in diabetic models [11]. *Bitter melon (Momordica charantia)* seed-enriched foods reduce hyperglycemia and improve metabolic parameters. Lotusine, a lotus alkaloid, mitigates hyperglycemia-induced oxidative stress by modulating the IRS-1/PI3K/Akt pathway [12]. The polyphenolic extract of *Acacia senegal (gum arabic)* also demonstrates antioxidant, antidiabetic, and cytoprotective effects [13]. Moreover, plant-derived compounds, including garlic (Allium sativum) and ginger (Zingiber officinale), have shown promise in diabetes management due to their antioxidant, anti-inflammatory, and metabolism-regulating properties [14, 15]. These findings underscore the importance of metabolic regulation, oxidative stress reduction, and modulation of signaling pathways in minimizing diabetes-related cellular damage. A related study found that a protein-based functional hydrogel significantly accelerated diabetic wound healing by improving the cellular microenvironment, regulating inflammation, enhancing angiogenesis, and promoting cutaneous nerve regeneration [16]. Therefore, strategies that include both nutritional and natural bioactive components and pharmacological approaches are gaining importance in diabetic wound healing. The agents investigated in this study differ from classical chalcone derivatives, as they are benzimidazolium–chalcone hybrid compounds that integrate two pharmacologically active structural motifs, benzimidazole and chalcone, within a single molecule. While the antidiabetic, antioxidant, and anti-inflammatory properties of chalcone derivatives have been extensively documented, research on the effects of these hybrid structures on fibroblast function, especially in the context of diabetic wound healing, remains limited [17, 18].

Although many treatments have been proposed to heal diabetic wounds, a global problem, only a few moderately effective compounds are currently applied clinically [7].Chalcones are a class of flavonoid precursors with diverse biological activities, including anti-inflammatory, antimicrobial, and pro-angiogenic effects, making them promising candidates for diabetic wound healing [14, 19]. Chalcones can suppress hyperglycemia by inhibiting α-glucosidase and α-amylase enzymes [20, 21]. They can reduce insulin resistance by activating peroxisome proliferator-activated receptor gamma (PPARγ) and reduce inflammation by inhibiting NFκB and cyclooxygenase-2 (COX-2) pathways [20]. In addition, they can increase the expression of antioxidant enzymes (Superoxide Dismutase (SOD), Catalase (CAT), Glutathione Peroxidase (GPx)) by activating the Nuclear factor erythroid 2–related factor 2 (Nrf2)/Antioxidant Response Element (ARE) pathway [22, 23]. It can exhibit strong antioxidant properties thanks to its ability to scavenge free radicals [24]. With these properties, various chalcone derivatives (methochalcone and sochalcone) have been approved as bile expectorant drugs and antiulcer agents, respectively, and have been reported to alleviate symptoms in clinical studies for chronic venous lymphatic insufficiency [25]. These natural compounds have several advantages: availability, ease of application, safe pharmacology, high efficacy, and affordable price [8]. In addition, chalcones are more stable and can be converted into promising bioactive scaffolds in medicinal chemistry [26]. Preclinical studies show that chalcones may be effective in treating diabetes and its complications through multiple mechanisms of action [17, 27]. A study reported that chalcone derivatives, 4-hydroxyderricin and xanthoangelol, significantly enhanced wound healing, particularly in endothelial cells. Another study suggested that chalcone, when combined with berberine, reduces oxidative stress and inflammation, increasing cell regeneration and thus promoting faster wound healing. Our study examined the therapeutic effects of newly synthesized chalcones on a glucose-induced in vitro wound model of diabetic fibroblasts, via multiple mechanisms.

## Materials & Methods

All reagents and solvents were obtained from commercial suppliers and used without further purification. NMR spectra were acquired on an Agilent 400 MHz FT-NMR spectrometer. High-resolution mass spectrometry (HRMS) was performed using an Agilent 6545 Accurate-Mass QTOF-MS instrument to determine the molecular masses. Reaction progress was monitored by thin-layer chromatography (TLC) on silica gel plates.

### Synthesis Procedure of Compound 1 (3-(4-(1 H-benzo[d]imidazol-1-yl)phenyl)−1-phenylprop-2-en-1-one)

A solution of potassium hydroxide (1.5 equiv.) in ethanol was added dropwise to an ethanolic solution containing equimolar amounts of 4-(1 H-benzo[d]imidazol-1-yl)benzaldehyde and acetophenone at room temperature. The reaction mixture was stirred for 12 h, during which a solid gradually precipitated. The resulting solid was isolated by filtration, washed with a 1:5 ethanol/water mixture, dried, and used in subsequent steps without further purification (yield: 83%) [28].

^**1**^**H-NMR (400 MHz**,** DMSO-*d***^***6***^**)** ***δ*** **(ppm)**: 8.64 (s, 1 H, Benzimidazole CH), 8.17 (dd, *J* = 11.1, 8.4 Hz, 4 H), 8.04 (d, *J* = 15.6 Hz, 1 H, CH = CH), 7.84 (d, *J* = 15.6 Hz, 1 H, CH = CH), 7.79 (dd, *J* = 7.8, 3.1 Hz, 3 H, Ar-H), 7.73–7.64 (m, 2 H, Ar-H), 7.59 (t, *J* = 7.6 Hz, 2 H, Ar-H), 7.34 (pd, *J* = 7.2, 1.4 Hz, 2 H, Ar-H). ^**13**^**C-NMR (100 MHz**,** DMSO-*d***^***6***^**)** ***δ*** **(ppm)**: 189.62, 144.42, 143.70, 143.26, 137.99, 137.98, 134.34, 133.72, 133.21, 131.07, 129.30, 129.05, 124.16, 124.13, 123.81, 123.20, 123.16, 120.52, 111.36, 39.98.

### Synthesis Procedure of Benzimidazolium-Chalcone Hybrid Compounds

Four separate reactions were performed to synthesize each target compound. For each reaction, 100 mg (0.3 mmol) of Compound 1 was weighed into a 50 mL round-bottom flask and dissolved in 10 mL of acetonitrile (CH₃CN) at 70 °C using a magnetic stirrer-heater. Subsequently, 0.3 mmol of the corresponding reagents—2-bromo-1-(4-nitrophenyl)ethan-1-one (2a), 2-bromo-1-(4-bromophenyl)ethan-1-one (2b), 2-bromo-1-(4-(trifluoromethyl)phenyl)ethan-1-one (2c), and 2-bromo-1-(naphthalen-2-yl)ethan-1-one (2d)—were added individually to the respective flasks. The reaction mixtures were stirred at 70 °C for 48 h. Upon completion of the reactions, as monitored by Thin Layer Chromatography (TLC), the solutions were allowed to cool to room temperature. The resulting precipitates were washed twice with diethyl ether, collected by centrifugation, and dried under vacuum to afford the pure products as C1-C4. All target molecules remained stable under ambient conditions and direct air exposure for a period of four months.

#### 3-(2-(4-nitrophenyl)−2-oxoethyl)−1-(4-(3-oxo-3-phenylprop-1-en-1-yl)phenyl)−1 H-benzo[d]imidazol-3-ium bromide (C1)

**Yield**: 85%. ^**1**^**H-NMR (400 MHz**,** DMSO-*d***^***6***^**)** ***δ*** **(ppm)**: 10.15 (s, 1 H, Benzimidazolium CH), 8.49 (d, *J* = 8.3 Hz, 2 H, Ar-H), 8.40 (d, *J* = 8.3 Hz, 2 H, Ar-H), 8.30 (d, *J* = 8.2 Hz, 2 H, Ar-H), 8.21 (t, *J* = 6.9 Hz, 3 H, Ar-H), 8.15 (d, *J* = 15.6 Hz, 1 H, CH = CH), 7.98 (t, *J* = 9.3 Hz, 3 H, Ar-H), 7.89 (d, *J* = 15.6 Hz, 1 H, CH = CH), 7.82–7.74 (m, 2 H), 7.70 (t, *J* = 7.1 Hz, 1 H, Ar-H), 7.60 (t, *J* = 7.5 Hz, 2 H, Ar-H), 6.57 (s, 2 H, ethylene -CH_2_-). ^**13**^**C-NMR (100 MHz**,** DMSO-*d***^***6***^**)** ***δ*** **(ppm)**: 190.69, 189.58, 151.17, 144.03, 142.45, 138.80, 137.78, 137.36, 134.60, 133.92, 132.43, 131.18, 131.09, 130.45, 129.35, 129.13, 128.16, 127.81, 126.09, 124.80, 124.60, 115.01, 114.24, 54.35. **HRMS (m/z)** [M-Br]^+^: calcd for C_30_H_22_N_3_O_4_^+^: 488.5225; found: 488.3038.

#### 3-(2-(4-bromophenyl)−2-oxoethyl)−1-(4-(3-oxo-3-phenylprop-1-en-1-yl)phenyl)−1 H-benzo[d]imidazol-3-ium bromide (C2)

**Yield**: 75%. ^**1**^**H-NMR (400 MHz**,** DMSO-*d***^***6***^**)** ***δ*** **(ppm)**: 10.14 (s, 1 H, Benzimidazolium CH), 8.29 (d, *J* = 8.4 Hz, 2 H, Ar-H), 8.22–8.14 (m, 3 H, Ar-H including CH = CH bond), 8.13–8.06 (m, 3 H, Ar-H), 7.97 (t, *J* = 9.3 Hz, 3 H, Ar-H), 7.93–7.84 (m, 3 H, Ar-H including CH = CH bond), 7.79–7.72 (m, 2 H, Ar-H), 7.69 (t, *J* = 7.3 Hz, 1 H, Ar-H), 7.59 (t, *J* = 7.5 Hz, 2 H, Ar-H), 6.47 (s, 2 H, ethylene -CH_2_-). ^**13**^**C-NMR (100 MHz**,** DMSO-*d***^***6***^**)** ***δ*** **(ppm)**: 190.67, 189.59, 144.06, 142.45, 137.79, 137.34, 134.62, 133.92, 133.21, 132.70, 132.45, 131.19, 131.07, 130.92, 129.36, 129.14, 128.13, 127.81, 126.06, 124.80, 114.94, 114.23, 53.89. **HRMS (m/z)** [M-Br]^+^: calcd for C_30_H_22_BrN_2_O_2_^+^: 522.4215; found: 523.2270.

#### 3-(2-oxo-2-(4-(trifluoromethyl)phenyl)ethyl)−1-(4-(3-oxo-3-phenylprop-1-en-1-yl)phenyl)−1 H-benzo[d]imidazol-3-ium bromide (C3)

**Yield: 74**%. ^**1**^**H-NMR (400 MHz**,** DMSO-*d***^***6***^**)** ***δ*** **(ppm)**: 10.21 (s, 1 H, Benzimidazolium CH), 8.37 (d, *J* = 7.9 Hz, 2 H, Ar-H), 8.31 (d, *J* = 8.0 Hz, 2 H, Ar-H), 8.20 (d, *J* = 7.7 Hz, 3 H, Ar-H), 8.15 (d, *J* = 15.6 Hz, 1 H, CH = CH), 8.07 (d, *J* = 8.0 Hz, 2 H, Ar-H), 7.99 (dd, *J* = 11.7, 8.4 Hz, 3 H, Ar-H), 7.89 (d, *J* = 15.6 Hz, 1 H, CH = CH), 7.82–7.73 (m, 2 H, Ar-H), 7.70 (t, *J* = 7.3 Hz, 1 H), 7.60 (t, *J* = 7.4 Hz, 2 H, Ar-H), 6.58 (s, 2 H, ethylene -CH_2_-). ^**13**^**C-NMR (100 MHz**,** DMSO-*d***^***6***^**)** ***δ*** **(ppm)**:190.96, 189.59, 144.06, 142.46, 137.79, 137.44, 137.36, 134.63, 134.29, 133.97, 133.92, 132.46, 131.20, 131.08, 129.88, 129.36, 129.15, 128.15, 127.82, 126.57, 126.53, 126.08, 125.49, 124.81, 122.78, 115.01, 114.25, 54.27. **HRMS (m/z)** [M-Br]^+^: calcd for C_31_H_22_F_3_N_2_O_2_^+^: 511.5237; found: 511.3077.

#### 3-(2-(naphthalen-2-yl)−2-oxoethyl)−1-(4-(3-oxo-3-phenylprop-1-en-1-yl)phenyl)−1 H-benzo[d]imidazol-3-ium bromide (C4)

**Yield**: 78%. ^**1**^**H-NMR (400 MHz**,** DMSO-*d***^***6***^**)** ***δ*** **(ppm)**: 10.26 (s, 1 H, Benzimidazolium CH), 9.00 (s, 1 H, Ar-H), 8.33 (d, *J* = 8.1 Hz, 2 H, Ar-H), 8.21 (ddd, *J* = 20.5, 14.4, 7.7 Hz, 6 H, Ar-H including CH = CH bond), 8.10 (dd, *J* = 8.2, 4.8 Hz, 2 H, Ar-H), 8.01 (dd, *J* = 13.3, 5.7 Hz, 3 H, Ar-H), 7.90 (d, *J* = 15.7 Hz, 1 H, CH = CH), 7.75 (ddt, *J* = 24.4, 13.3, 6.0 Hz, 5 H, Ar-H), 7.61 (t, *J* = 7.6 Hz, 2 H, Ar-H), 6.66 (s, 2 H, ethylene -CH_2_-). ^**13**^**C-NMR (100 MHz**,** DMSO-*d***^***6***^**)** ***δ*** **(ppm)**: 191.19, 189.56, 144.15, 142.51, 137.76, 137.30, 136.10, 134.69, 133.96, 132.55, 132.52, 131.57, 131.43, 131.23, 131.07, 130.23, 129.97, 129.38, 129.28, 129.16, 128.42, 128.13, 127.97, 127.83, 126.06, 124.70, 123.81, 114.96, 114.27, 53.98. **HRMS (m/z)** [M-Br]^+^: calcd for C_34_H_25_N_2_O_2_^+^: 493.5855; found: 493.3280.

**Fig. 1:**
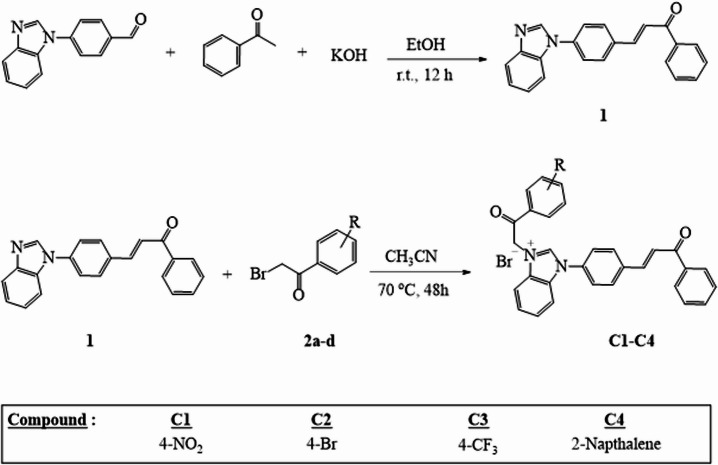
Synthesis procedure of benzimidazoium-chalcone hybrid derivatives

The synthesis scheme of benzimidazolium-chalcone hybrid compounds is given in Fig. 1.

### Cell Culture and Experimental Groups

The human dermal fibroblast cell line (HDF-1), a crucial component of our experiments, was purchased from Atlas Biotechnology. HDFs were maintained in Dulbecco’s Modified Eagle Medium (DMEM) (Wisent Inc. 319-013-CL) supplemented with 10% fetal bovine serum (FBS), 2% L-glutamine, and 1% penicillin-streptomycin (Gibco). For the diabetic group, cells were incubated with D-glucose (Merck) (50 mM) for 72 h [29, 30].

### α-Glucosidase Activity Assay

The α-glucosidase inhibitory activity was evaluated according to our previously reported method. The inhibitory potentials of compounds bearing different substituents were assessed at concentrations ranging from 0.5 to 2 µg/mL. Briefly, test samples at various concentrations were added to the enzyme solution prepared in phosphate buffer. Following incubation, the substrate α-D-glucopyranoside was introduced, and absorbance was measured at 405 nm using a spectrophotometer. Each experiment was performed in triplicate. Acarbose was used as positive control [31].

### 3-(4,5-Dimethylthiazol-2-yl)−2,5 − 2,5-Diphenyltetrazolium Bromide (MTT) Assay

The MTT assay, a well-established protocol, uses a tetrazolium salt that is reduced by mitochondrial metabolism in living cells, forming a purple formazan product. The MTT kit (Cayman) was used to evaluate the toxicity of chalcone compounds. HDF-1 cells were seeded at a density of 2.5 × 10^4^ cells/well in a 96-well plate and incubated overnight. After 24 h, cells were treated with chalcone (1 µM, 5 µM, 25 µM, 50 µM, 100 µM) compounds for 24 h. After the indicated time points, the culture medium was removed for the MTT assay, and 20 µL of MTT solution (5 µg/mL) was added to each well. The plates were then incubated at 37 °C for 4 h, and after removing the MTT solution, 100 µL of DMSO (Sigma) was added to dissolve formazan crystals. Absorbance was measured at 570 nm using a microplate reader (Thermo Scientific Multiskan Go) [8].

### Wound Scratch Assay

High-glucose environments are a standard approach for simulating diabetes in in vitro studies and are widely used to mimic a diabetic environment. Therefore, in our study, a diabetic wound model was created using 50 mM D-glucose for 72 h [4]. HDF-1 cells were seeded in a 12-well plate. Then, the IC_50_ values of all compounds were determined in diabetic HDF-1 cells. After 48 h, a “scratch” was created with a 200-µL pipette tip. Fibroblast migration was photographed at 0, 24, and 48 h using an inverted microscope equipped with a digital camera (Euromex) to ensure data accuracy. The ImageJ software was used to measure the scratch area [32]. Wound closure percentage was calculated based on the initial wound area and the wound area at a specific time point: 24 h wound healing rate = (0 h distance-24 h distance)/0 h distance × 100, and 48 h wound healing rate = (0 h distance-48 h distance)/0 h distance × 100.

### Total Antioxidant Status (TAS) Measurement

Adhering strictly to the manufacturer’s instructions, we determined TAS levels in HDF-1 cells using the TAS Colorimetric Assay Kit (Elabscience, E-BC-K801-M). Trolox was prepared at different concentrations as a positive control and used as a reference standard for TAS measurements. The TAS values ​​in our samples were then calculated as mmol/L, ensuring the validity of our Method [14].

### Type I Procollagen and Cytokine Analysis

Type I procollagen (BTB-E1385Hu), TNF-α (BT Lab, E0082Hu), and IL-1β (BT Lab, E0119Ra) levels were quantified using an enzyme-linked immunosorbent assay (ELISA). The analysis was performed according to the instructions provided with the kits. The absorbance of all groups was measured at 450 nm using a microplate reader (Thermo Scientific Multiskan Go) [33].

### Ki67, NFκB, PDGFA Immunostain

HDF-1 cells grown on coverslips were fixed with 4% paraformaldehyde, permeabilized with 0.1% Triton X-100, and endogenous peroxidase activity was blocked using 3% H₂O₂. After blocking, cells were incubated overnight at 4 °C with Ki67 (bs-2310R), NFκB (bs-10037R), and PDGFA (bs-0196R) primary antibodies (Bioss Antibodies), followed by HRP-conjugated secondary antibodies. Immunoreactivity was visualized using DAB and counterstained with hematoxylin [34, 35]. Immunoreactivity was assessed semi-quantitatively using the H-score system, which incorporates both staining intensity and the percentage of positive cells. The staining intensity (scored 0 to 3) was multiplied by the proportion of positive cells (scored 0 to 4) to yield a total score ranging from 0 to 12 [36].

### Molecular Docking

Molecular docking was performed using Maestro (version 13.8). The chemical structures of the synthesized compounds were prepared using ChemDraw. The three-dimensional structure of the α-glucosidase–related protein (PDB ID: 5NN8) was obtained from the Protein Data Bank. After protein preparation, a receptor grid box with dimensions of 20 × 20 × 20 Å was generated to define the active site. Docking results were evaluated and expressed as binding energies (kcal/mol) [14, 37–39].

### Statistical Analysis

Experimental data are reported as mean ± standard deviation (SD) from three independent experiments (*n* = 3). One-way ANOVA assessed differences between groups for each biological endpoint. Post hoc tests compared treatment groups with the diabetic group, with significance set at *p* < 0.05. For direct comparisons between treatment groups, pairwise analyses used Bonferroni post hoc tests, depending on normality and homogeneity of variance. All statistical analyses and graphs were produced using GraphPad Prism 9.0 (GraphPad Software, San Diego, CA, USA).

## Result

### Chemistry

The synthetic routes for Compound 1 and the benzimidazolium–chalcone hybrid derivatives are illustrated in Fig. 1. Compound 1 was obtained by reacting the corresponding aldehyde and acetophenone at room temperature for 30 min. Compound 1 was dissolved in acetonitrile to prepare the quaternary ammonium salts and reacted with the appropriate benzyl bromide derivatives. Four novel products were isolated as pure precipitates. The structures of all synthesized compounds were confirmed by ^1^H and ^13^C NMR spectra, acquired on an Agilent 400 MHz FT-NMR spectrometer. Additionally, high-resolution mass spectrometry (HRMS) was performed using an Agilent 6545 Accurate-Mass QTOF-MS instrument to determine the molecular masses.

### Spectroscopic Analysis of the Compounds

The ¹H NMR spectrum of Compound 1 (Fig. S1) exhibited a singlet at 8.64 ppm, attributed to the benzimidazole CH proton. The aromatic and other proton resonances were observed within the expected chemical shift range of 7.31–8.19 ppm. In addition, the conjugated alkene fragment should exhibit characteristic signals in the 1 H NMR spectrum. The corresponding protons are expected to appear as doublets with a coupling constant of approximately J ≈ 16 Hz (consistent with a trans-configuration). The ¹H NMR spectra of the benzimidazolium–chalcone derivatives (C1–C4) (Supplement file) (Figs. S3, S6, S9, S12) displayed singlet signals near 6.5 ppm, corresponding to the methylene (–CH₂–) protons, with each signal integrating for two protons. For all derivatives, aromatic and olefinic (–CH = CH–) proton signals were observed in the range of 7.57–8.50 ppm. The benzimidazolium CH proton appeared as a distinct resonance around 10 ppm.

In the ¹³C NMR spectrum of Compound 1 (Fig. S2), the carbonyl (C = O) carbon appeared at 189.62 ppm, while aromatic carbons resonated between 111.36 and 144.42 ppm. In the ¹³C NMR spectra of the benzimidazolium–chalcone hybrid salts (C1-C4), signals appearing near 50 ppm were assigned to the methylene (–CH₂–) carbons (Figs. S4, S7, S10, S13). Aromatic and olefinic carbon resonances were observed across the range of 114.23–151.17 ppm for all compounds. The carbonyl (C = O) carbons adjacent to the –CH = CH– moiety appeared around 189 ppm, while additional carbonyl signals, associated with the benzimidazole ring, were detected near 190 ppm in each compound.

In addition, high-resolution mass spectrometry (HRMS) analysis of all compounds was consistent with the calculated molecular masses (Figs. S5, S8, S11, S14).

The comprehensive spectroscopic data conclusively confirm the successful synthesis of the target compounds.

### α-Glucosidase Activity Assay

The α-glucosidase inhibitory potentials of the compounds prepared at different concentrations were investigated. According to the results, the IC₅₀ values ranged from 1.115 to 1.557 µg/mL. Among the tested compounds, compound C4 exhibited the strongest inhibitory activity. Acarbose was used as the positive control, and its IC₅₀ value was calculated as 3.503 µg/mL (Table 1). These findings demonstrate that the synthesized compounds act as more potent α-glucosidase inhibitors than the positive control.

**Table 1:** IC_50_ values of C1, C2, C3 and C4 against α-Glucosidase

Compounds	Regression equation	*R*^2^	IC_50_ (µg/mL)
C1	y = −0.2788x + 0.8792	0.9859	1.557
C2	y = −0.3578x + 0.8586	0.9914	1.156
C3	y = −0.2544x + 0.8552	0.9554	1.612
C4	y = −0.356x + 0.842	0.98	1.115
Acarbose	y = −0.13x + 0.9004	0.9897	3.503

### Cell Viability

**Fig. 2:**
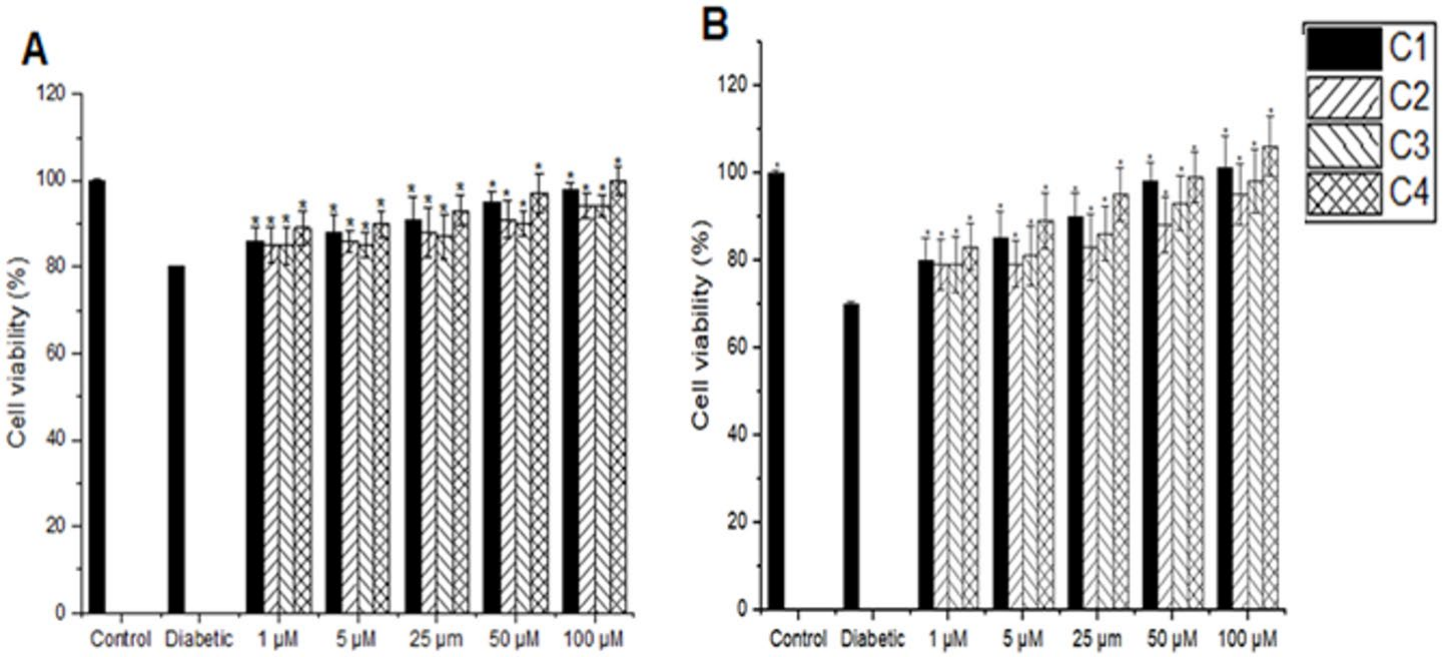
Diabetic HDF-1 cell viability graph treated with compounds C1, C2, C3, and C4 for 24 (**A**) and 48 h (**B**). Control: D-glucose and compounds were not added. All values ​​are shown as the mean ± SD of 3 independent experiments (*n* = 3). *When the groups were compared with the diabetic group, *p* < 0.05

Compounds applied to HDF-1 cells at concentrations of 1 µM (C1, C2, C3, C4: 86.3 ± 3.1, 85.3 ± 4.2, 85 ± 5.3, 89.6 ± 6.1 respectively), 5 µM (88 ± 6.2, 86.2 ± 2.5, 85.9 ± 2.8, 90.2 ± 3.1 respectively), 25 µM (91.2 ± 5.3, 88.4 ± 6.7, 87.1 ± 8.2, 93.2 ± 3.4 respectively), 50 µM (95.7 ± 2.3, 91.4 ± 3.1, 90.5 ± 2.8, 97.6 ± 4.4 respectively), and 100 µM (98.6 ± 2.5, 94.4 ± 2.9,94.1 ± 2.5, 99.7 ± 3.5 respectively), did not reduce cell viability (%) at 24 h compared to the control group (100 ± 0.3) (Fig. 2A). At the end of 24 h of incubation, HDF-1 cells treated with various concentrations of C1 and C4 (50–100 µM) were similar to the control group (Fig. 2A) (*p* > 0.05). Also, HDF-1 cells treated with C2 and C3 showed similar cell viability (Fig. 2A) (*p* > 0.05). In the MTT assay, HDF-1s treated with compounds C1, C2, C3, and C4 at 48 h (1 µM: 80.2 ± 5.9, 79.5 ± 5.7, 79.3 ± 6.3, 83.7 ± 5.2, 5 µM: 85.4 ± 6.1, 81.8 ± 5.4, 79.5 ± 6.8, 89.6 ± 6.4, 25 µM: 90.1 ± 5.3, 86.5 ± 7.6, 83.6 ± 6.2, 95.2 ± 6.1, 50 µM: 98.4 ± 4.3, 93.4 ± 6.2, 88.2 ± 6.1, 99.1 ± 6.5, 100 µM: 101.2 ± 7.5, 98.5 ± 6.9, 95.7 ± 7.3, 106.1 ± 6.8 respectively) of incubation showed increased cell viability (%), compared to the diabetic group (70.3 ± 0.9) (Fig. 2A and B) (*p* < 0.05). In diabetic HDF-1 cells, after 48 h of incubation, all compounds had approximately 80% viable cells at the lowest experimental concentration 1–5 µM (Fig. 2B) (*p* < 0.05). This indicates that the compounds exhibit a remarkably nontoxic.

### Migration Assay

**Fig. 3:**
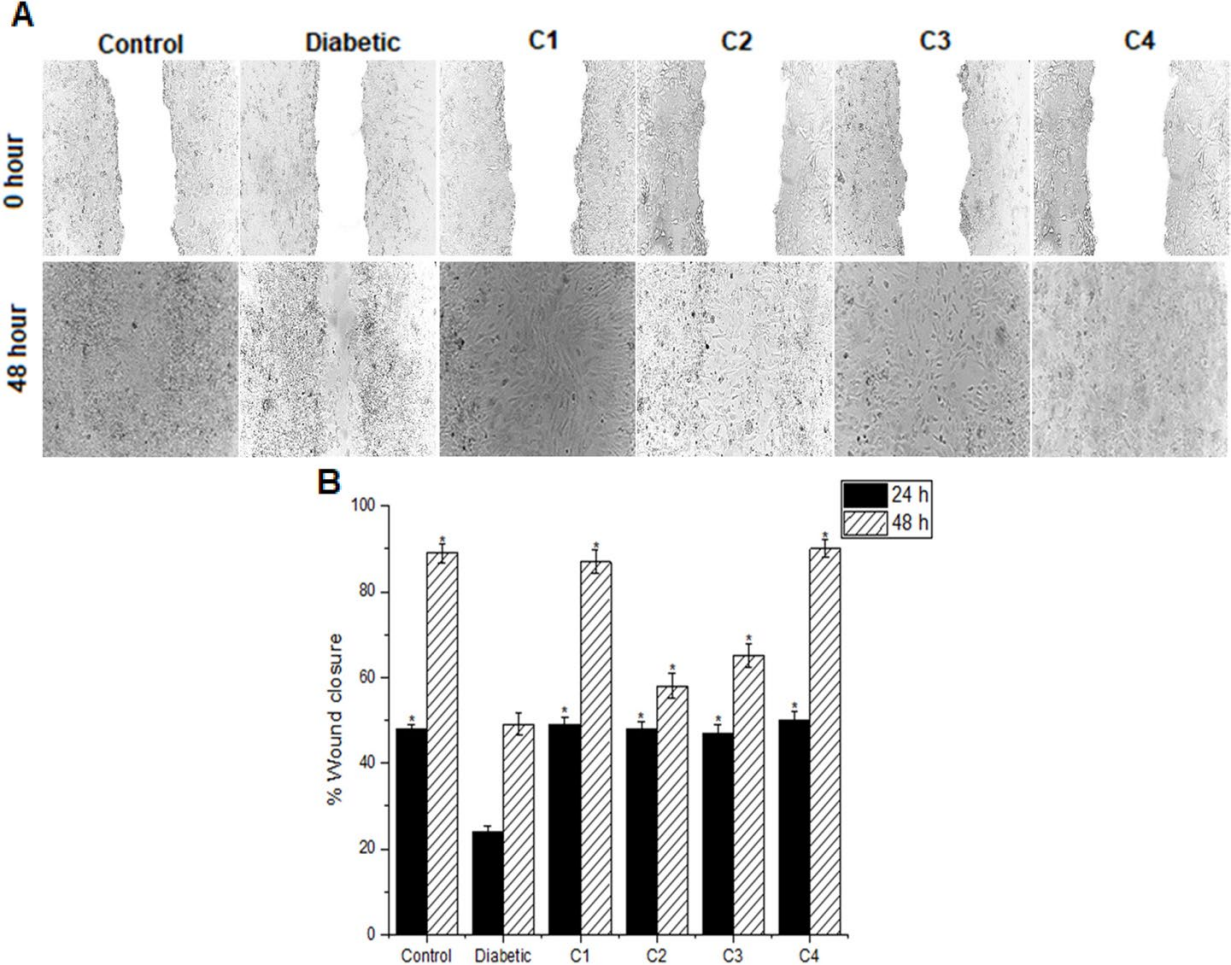
Microscopic image of diabetic HDF-1 cell migration (**A**) and % wound closure rate (**B**) treatment with C1, C2, C3, and C4. All values ​​are shown as the mean±SD of 3 independent experiments (n = 3). *When the groups were compared with the diabetic group, p<0.05

In the scratch analysis, there was a wound healing percentage of 48.8% in the control group at 24 h, while all treatment groups (49.3 ± 1.5, 48.2 ± 1.6, 47.7 ± 1.9, and 50.4 ± 2.1 respectively) were similar to the control group (48.1 ± 1.1) (Fig. 3B) (*p* > 0.05). There was a wound healing percentage of 89.7 ± 2.3 in the control group at 48 h, while compounds C1 and C4 (87.2 ± 2.8 and 90.3 ± 2.1 respectively) were similar to the control group (Fig. 3 A) (*p* > 0.05). However, as shown in Fig. 3, the wound-healing percentages of groups C2 and C3 at 48 h (58.6 ± 3.1 and 66.2 ± 2.8 respectively) were lower than those of the control group (*p* < 0.05). The diabetic group exhibited wound-healing percentages of 49.4 ± 2.6 at 48 h, which were significantly lower than those of all treatment groups (Fig. 3B) (*p* < 0.05).

### TNF-α, IL-1β, Procollagen Type I, and TAS Levels

**Fig. 4:**
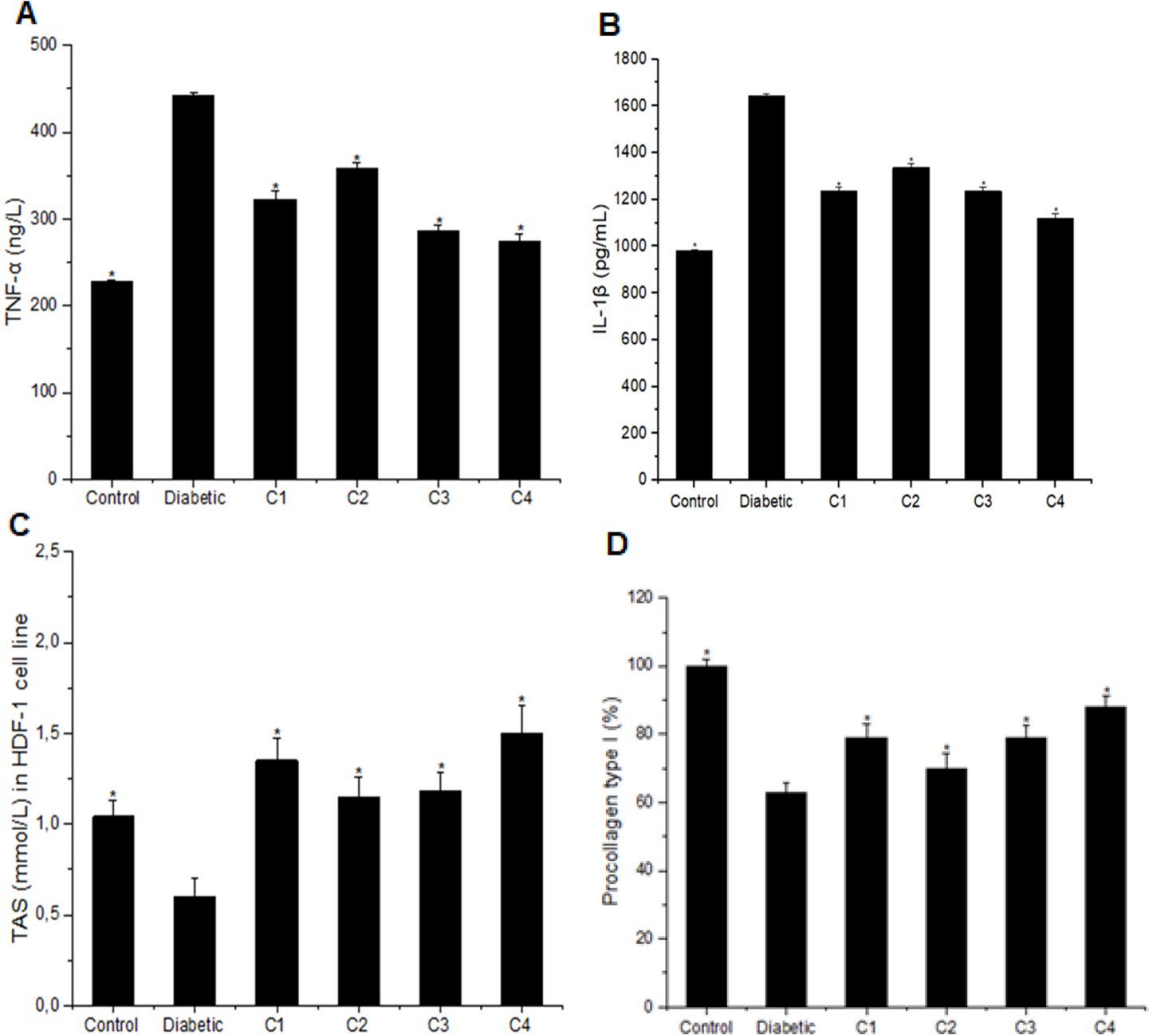
Comparative graphs showing the TNF-α (**A**), IL-1β (**B**), TAS (**C**), and procollagen type I levels (**D**) in diabetic HDF-1 cells treated with C1, C2, C3, and C4. All values​​are shown as the mean±SD of 3 independent experiments (n = 3). *When the groups were compared with the diabetic group, p<0.05

TNF-α and IL-1β levels were 227.2 ± 2.2 and 979.2 ± 7.3 respectively, in the control group. Following chalcone treatment of HDF-1 cells treated with D-glucose, C4, in particular, reduced TNF-α (C1, C2, C3, C4: 322.1 ± 7.2, 358.6 ± 6.1, 285.4 ± 5.3, 273.7 ± 6.8 respectively) and IL-1β (C1, C2, C3, C4: 1236.3 ± 16.6, 1335.2 ± 15.3, 1233.4 ± 18.3, 1118.2 ± 19.3 respectively) levels compared to the diabetic group (TNF-α: 442.4 ± 5.3, IL-1β: 1642.4 ± 8.6) (Fig. 4 A and B) (*p* < 0.05). Glucose disrupts the oxidative balance in HDF-1 cells, as evidenced by a decreased antioxidant capacity (Fig. 4 C) (*p* < 0.05). However, after treatment with all compounds, TAS (C1, C2, C3, C4:1.35 ± 0.12, 1.15 ± 0.10, 1.18 ± 0.11, 1.5 ± 0.15 respectively) and procollagen type I (C1, C2, C3, C4: 79.2 ± 1.9, 70 ± 2.3, 79.4 ± 2.6, 88.3 ± 2.1 respectively) levels in HDF-1 cells were observed to increase compared to the diabetic group (TAS: 0.6 ± 0.10, procollagen type I: 63.1 ± 0.8) (Fig. 4 C and D) (*p* < 0.05). Additionally, TAS levels in diabetic cells treated with C1 and C4 were higher than in the control group (1.04 ± 0.09) (Fig. 4 C) (*p* < 0.05). On the other hand, type I procollagen levels in treated diabetic HDF-1 cells were lower than in the control group (100.2 ± 0.9) (Fig. 4D) (*p* < 0.05).

### Immunolabeling of Ki67, NFκB, PDGFA

**Fig. 5:**
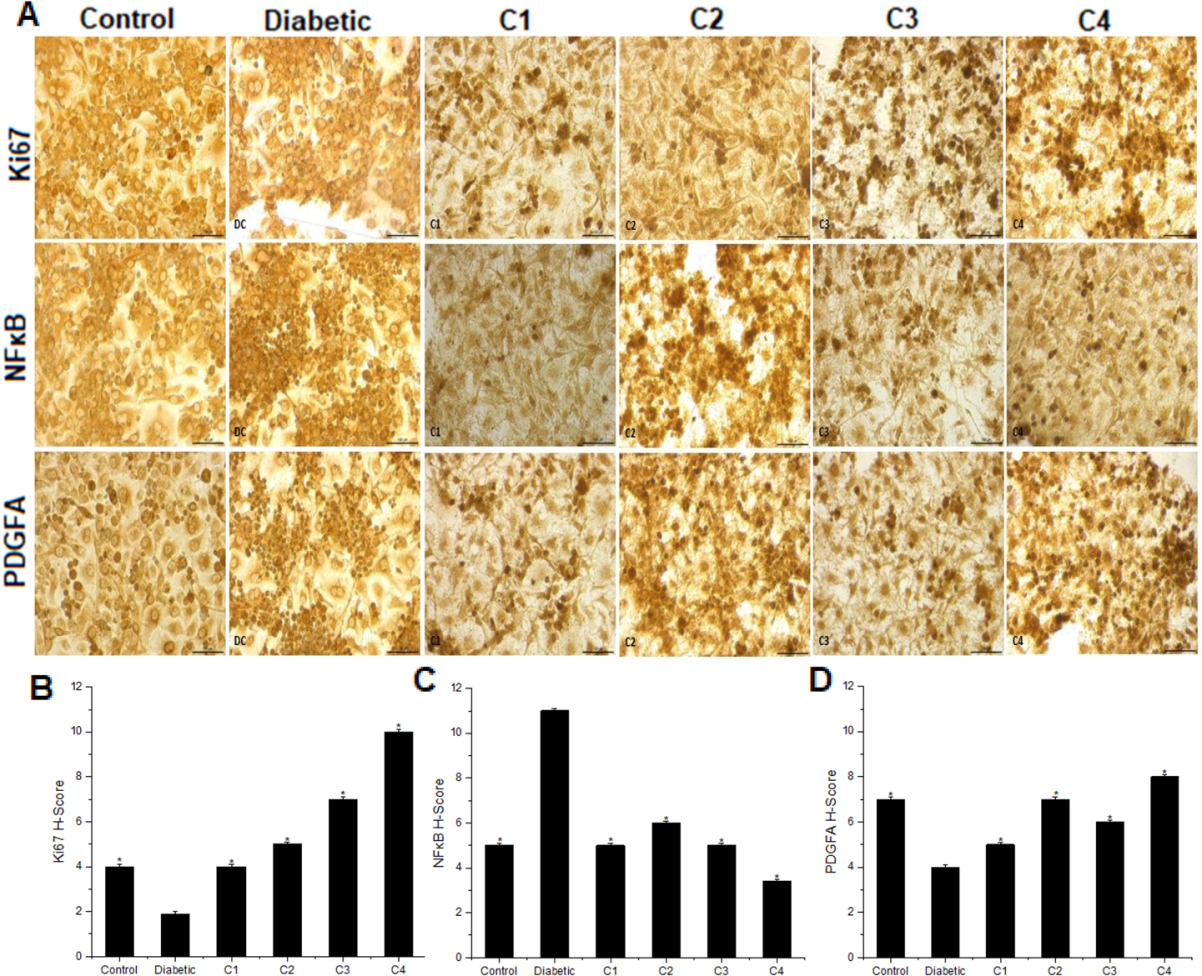
Representative histological image of diabetic HDF-1 cells following treatment with C1, C2, C3, and C4, and subsequent immunostaining for Ki67, NFκB, and PDGFA. Scale bars: 100 μm (**A**). H-score graph of Ki67 (**B**), NFκB (**C**), and PDGFA (**D**) immunoreactivity (mean±SD). *When the groups were compared with the diabetic group, *p*<0.05

According to the H-score evaluation, the treatment groups showed a statistically significant increase in Ki67 nuclear and PDGFA cytoplasmic immunoreactivity compared to the diabetic group (*p* < 0.05) (Fig. 5A and B, and 5D). In contrast, NFκB nuclear immunoreactivity H-scores were significantly decreased in the treatment groups relative to the diabetic group (*p* < 0.05) (Fig. 5A and C). These results indicate that compounds C1, C2, C3, and C4 may suppress NFκB activity while enhancing cellular proliferation and growth factor expression.

### Molecular Docking

Among the synthesized compounds, compound C4 exhibited the strongest α-glucosidase inhibitory activity and was therefore selected for molecular docking analysis to investigate its interaction with the 5NN8 α-glucosidase protein. According to the docking results, compound 2098 showed a binding energy of − 5.051 kcal/mol. Although experimental data indicated that compound 2098 was more potent than the positive control acarbose, the docking score of acarbose was calculated as − 7.168 kcal/mol (Table 2).

**Table 2:** Molecular docking scores and Glide emodel values

Compounds	Protein	Docking score	Glide e model
**C4**	5NN8	−5.051	−63.330
**Acarbose**	5NN8	−7.168	−92.530

**Fig. 6:**
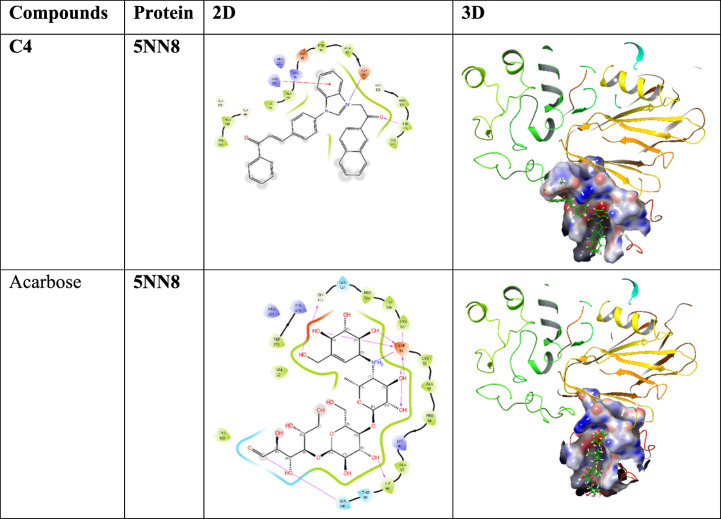
2D and 3D structural representations of the target proteins

Interaction analysis revealed that the benzene ring of the benzimidazole moiety forms a π–cation interaction with ARG331. Additionally, the carbonyl group in the structure forms a hydrogen bond with TRP126, while the positively charged nitrogen (N⁺) participates in a salt bridge interaction with ASP91. These interactions stabilize compound 2098 within the enzyme’s active site (Fig. 6).

## Discussion

Wounds in diabetic patients are the leading cause of non-traumatic amputations, with infection accounting for an average mortality rate of 50% [6]. Effective modulation of wound healing is therefore essential. This process includes hemostasis, inflammation, debridement, proliferation, epithelialization, and remodeling [40, 41], but its molecular and cellular regulation remains incompletely understood. Chronic wounds present a major global healthcare challenge and result in significant medical costs [8]. As a result, new strategies to accelerate wound healing are being explored. Chalcones have demonstrated antidiabetic properties in several studies. They may reduce insulin resistance by regulating adipogenesis via peroxisome proliferator-activated receptor gamma (PPAR-γ) [17], enhance glucose uptake by increasing GLUT4 expression in muscle and adipose tissue, and protect pancreatic β-cells by reducing oxidative stress and chronic inflammation associated with diabetes pathogenesis [17, 42]. This study investigates the effects of newly synthesized chalcones on wound healing in HDF-1 cells under high-glucose conditions, focusing on inflammation, growth factor signaling, antioxidant mechanisms, and cell proliferation.

Chalcones (1,3-diaryl-2-propen-1-ones), as biosynthetic precursors of flavonoids, stand out for their potent antidiabetic properties [43]. These compounds may accelerate healing by increasing fibroblast proliferation in diabetic wound models. One study suggested that various chalcone derivatives increase fibroblast proliferation in vitro and in vivo [44]. Our study confirms increased growth rates in diabetic fibroblasts treated with C1, C2, C3, and C4 compared with untreated diabetic fibroblasts, as observed by Salehi et al. (Figure 2A and B) (*p* < 0.05) [17]. Diabetic wound fibroblasts are likely impaired due to cell senescence associated with or induced by diabetes (intrinsic senescence) and the wound environment [45]. Given that glucose inhibits microtubule formation at high concentrations, we believe that a defect in microtubule structure, which is effective in cell proliferation, may contribute to the intrinsic mechanism involved in the decreased cell proliferation observed in diabetic wound fibroblasts [14, 46]. Therefore, the increased intensity of Ki67 proliferation marker and growth factor immunostaining in the treatment groups compared to the diabetic group, and the correlation of this increase with fibroblast proliferation, support the potential effectiveness of chalcone compounds in this process. Furthermore, effective compound doses were determined using alpha-glucosidase inhibition results. At these concentrations, fibroblast metabolic activity remained stable, while cell proliferation increased. These results demonstrate the compounds’ non-toxicity and support their potential for therapeutic application.

Dermal fibroblasts (DF) play a key role in wound healing by proliferating and migrating into the wound bed to form granulation tissue [47]. Previous studies in mice have indicated that the migratory ability of diabetic DFs is impaired, with diabetic DFs migrating 75% less than normal DFs. Recently, a novel naphthalcone derivative, 2-(5-(2,4,6-trimethoxyphenyl)−4,5-dihydro-1 H-pyrazol-3-yl)naphthalen-1-ol (TDPN), demonstrated significant activity in promoting the migration and proliferation of HaCaT cells [48]. In this study, glucose inhibited DF migration, but chalcone compounds reversed this effect (Fig. 3 A and B) (*p* < 0.05). Compound C4, which contains a 2-naphthalene ring, demonstrated significantly greater biological activity than chalcones with 4-NO₂, 4-Br, or 4-CF₃ groups (Figs. 1 and 3 A and B) (*p* < 0.05). This enhanced activity was consistent across cell migration and proliferation analyses. Chalcone derivatives with a 2-naphthalene ring are known for high lipophilicity, increased membrane permeability, and strong hydrophobic and π–π interactions with intracellular signaling proteins which likely contribute to C4’s superior activity. These features also support more efficient activation of signaling pathways involved in cytoskeletal remodeling and cell cycle progression, accelerating migration and proliferation [49, 50]. These properties are associated with C4’s enhanced biological activity. Additionally, these characteristics facilitate more efficient activation of signaling pathways involved in cytoskeletal remodeling and cell cycle progression, thereby accelerating cell migration and increasing proliferation. Other studies have reported that different chalcone derivatives substantially affect the cell cycle and migration of human breast and lung cancer cells, suggesting that the effects of chalcone derivatives on cell migration may vary with structural properties, concentration, and cell type [51]. In another study, the potent antioxidant properties of chalcone compounds were associated with increased fibroblast cell migration [52]. Chalcone compounds may reduce cell damage by scavenging free radicals and regulate fibroblast proliferation and migration by affecting the cell cycle. On the other hand, cell migration is regulated by various signaling pathways, including MAPK and NFκB [48]. High glucose (hyperglycemia) causes oxidative stress and chronic activation of the NFκB pathway in fibroblast cells, and increases IL-1β production [53]. Consequently, fibroblast migration and proliferation are reduced, and wound healing is delayed or suppressed. Indeed, in our study, the decrease in NFκB immunostaining intensity and TNF-α, IL-1β levels, but an increase in TAS, in the treatment groups compared to the diabetic group, indicates inhibition of the NF-κB pathway by chalcone compounds (Fig. 4 A and B, 4 C, and Fig. 5A and C) (*p* < 0.05). This inhibition is significant because it suggests that chalcone compounds may counteract the adverse effects of hyperglycemia on the NFκB pathway, thereby promoting fibroblast migration and wound healing. Furthermore, it was concluded that PDGFA activation modulated cell migration by suppressing NFκB. Indeed, a higher PDGFA H-score was observed in the treatment groups compared to the diabetic group (Fig. 5A and C) (*p* < 0.05). (Figure 5A and C) (*p* < 0.05).

During the inflammatory phase of wound healing, DF secrete cytokines, chemokines, and growth factors that coordinate immune cell migration and support cell survival in damaged tissue [54]. PDGFA is essential for stimulating fibroblast chemotaxis and proliferation [55, 56]. In diabetes, elevated NFκB and IL-1β levels impair PDGFA and TGF-β activation, reducing fibroblast migration [57]. The chalcone treatment in this study shows promise by inducing PDGFA expression, which may enhance fibroblast migration through improved growth factor signaling. This effect is associated with reduced inflammation and increased antioxidant activity, mediated by α-glucosidase inhibition. The reactivation of these growth factors suggests that the compounds lower intracellular oxidative stress through their antioxidant properties. Structural analogs of flavones have been shown to promote procollagen production in DF and provide anti-aging effects [14, 48]. In this study, higher PDGFA levels in the treatment groups directly correlated with increased type I procollagen production, the main structural protein in new connective tissue (Figs. 4D and 5A and D) (*p* < 0.05). The results indicate that the biological activity of chalcone derivatives depends on both their core scaffolds and substituents. Compound C4, which contains a 2-naphthalene ring, showed greater activity than other derivatives (C1: 4-NO₂, C2: 4-Br, C3: 4-CF₃). This enhanced activity is likely due to the large, conjugated naphthyl group in C4, which expands the π-electron system and enables stronger π-π interactions with intracellular target proteins. The naphthalene structure also increases lipophilicity, improving membrane permeability and bioavailability, and may enhance binding affinity through stronger interactions with hydrophobic protein regions [58–60]. These features likely contribute to more effective modulation of NF-κB, MAPK, and growth factor signaling pathways involved in cell migration and proliferation. As a result, C4 was identified as the most potent compound for promoting fibroblast proliferation, migration, and anti-inflammatory activity.

Recent research increasingly focuses on reprogramming cellular metabolism rather than treating only the symptoms of diabetes-related complications [61]. In this context, cyanoglycosides derived from Moringa oleifera seeds have been reported to attenuate the progression of diabetic nephropathy by inhibiting the PFKFB3/TGF-β1/SMAD signaling axis, thereby restoring metabolic homeostasis [62]. These findings emphasize that targeting metabolic dysregulation represents a critical strategy for mitigating diabetes-associated tissue damage. In parallel, the chalcone derivatives evaluated in this study showed strong anti-inflammatory and cytoprotective effects, as well as improved fibroblast function and growth factor signaling. These benefits are likely mediated, at least in part, by modulating cellular stress responses. This suggests that chalcones may help restore redox balance and metabolic integrity in hyperglycemic conditions. Chalcones are naturally occurring flavonoids found in plant sources such as licorice root, apple peel, and various flowers. They are well documented for their anti-inflammatory, antioxidant, and antimelanogenic activities [63]. Several chalcone derivatives disrupt key inflammatory signaling pathways. For example, methyl-1-hydroxy-2-naphthoate suppresses lipopolysaccharide-induced inflammation in macrophages by inhibiting the NFκB, JNK, and p38 MAPK pathways [64]. Similarly, lycochalcone A reduces inflammation by downregulating NFκB activation and prostaglandin E2 (PGE2) production [17]. Consistent with these reports, our findings showed a significant reduction in IL-1β and NFκB expression in diabetic fibroblasts after chalcone treatment (Figs. 4B and 5A and C; *p* < 0.05), supporting the hypothesis that these compounds suppress pro-inflammatory signaling. Additionally, the upregulation of growth factor expression and the increase in total antioxidant status (TAS) indicate that chalcones not only reduce inflammation but also promote cellular repair and strengthen antioxidant defenses. Emerging nanotechnological approaches highlight the need to target inflammation, oxidative stress, and metabolic imbalance in diabetic wound healing [65–67]. Aggregation-induced emission (AIE) luminogen-based nanostructures that release carbon dioxide can accelerate tissue regeneration by suppressing inflammation, reducing oxidative stress, and enabling real-time bioimaging [68]. Similarly, glucose-responsive hydrogels loaded with nanozymes offer a multifunctional platform for regulating local glucose levels, providing antibacterial activity, and supporting tissue repair [69]. The biological effects of chalcone derivatives observed in this study are similar to those achieved with these advanced nanotechnologies. This suggests that chalcones may offer a promising, more accessible therapeutic option for modulating key molecular pathways involved in diabetic tissue repair.

## Conclusion

This study comprehensively evaluated the effects of newly synthesized chalcone derivatives on wound healing in a diabetic wound model under high glucose conditions. The results demonstrated that chalcone compounds promoted proliferation and migration in diabetic fibroblasts, suppressed inflammation, and reduced oxidative stress. Inhibition of the NF-κB signaling pathway, reductions in IL-1β and TNF-α levels, and increases in total antioxidant capacity provided evidence for the anti-inflammatory and antioxidant effects of these compounds. Additionally, increased PDGFA expression and higher Ki67 staining intensity indicated enhanced growth factor-mediated tissue repair and cell proliferation. Although chalcone derivatives exhibited relatively low inhibition of α-glucosidase, this effect does not appear to be the primary mechanism in wound healing. The principal effects are mediated through suppression of inflammation, reduction of oxidative stress, and improvement of cellular functions. These findings suggest that chalcones exert a multitarget mechanism of action.

Evaluation of the structure-activity relationship indicates that the higher biological activity of compound C4, which contains a 2-naphthalene ring, compared to other derivatives can be attributed to its extensive π-conjugation, increased lipophilicity, and enhanced capacity to interact with intracellular targets. These properties enable C4 to more effectively suppress cell proliferation, migration, and inflammation. In particular, compound C4 stands out as a promising candidate for further in vivo studies and clinical trials due to its strong biological activity. These results provide a translational basis for developing more effective and versatile strategies for diabetic wound treatment.

## Supplementary Information

Supplementary Material 1.

## Data Availability

The datasets used and/or analyzed during the current study are available from the corresponding author upon reasonable request.
