# Supplementary material for: In Vitro Evaluation of Novel Synthetic Benzimidazolium–Chalcone Hybrids: Antioxidant and Regenerative Effects in Diabetic Wound Healing
Source: Appl Biochem Biotechnol. 2026 May 8;198(8):5873–94. doi: 10.1007/s12010-026-05712-y (PMC13407772; doi:10.1007/s12010-026-05712-y)
Supplement: Supplementary file 1 — Supplementary Material 1. [file 12010_2026_5712_MOESM1_ESM.docx]

**Effects of New Synthetic Benzimidazolium-Chalcone Hybrid Derivatives on Antioxidant, Proliferative, Anti-Inflammatory and Growth Factors in In Vitro Diabetic Wound Healing**


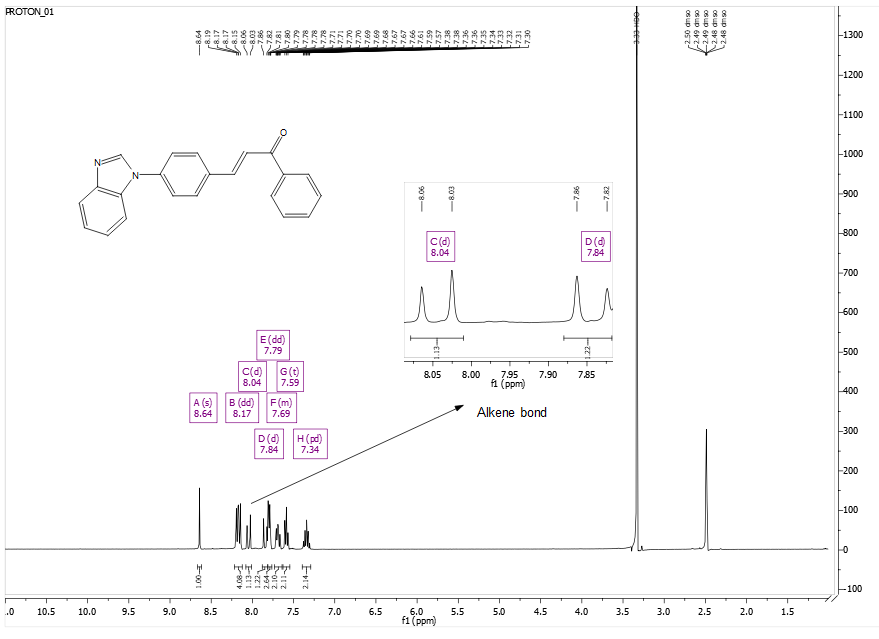
**Fig. S1.** Compound 1 ^1^H-NMR spectra


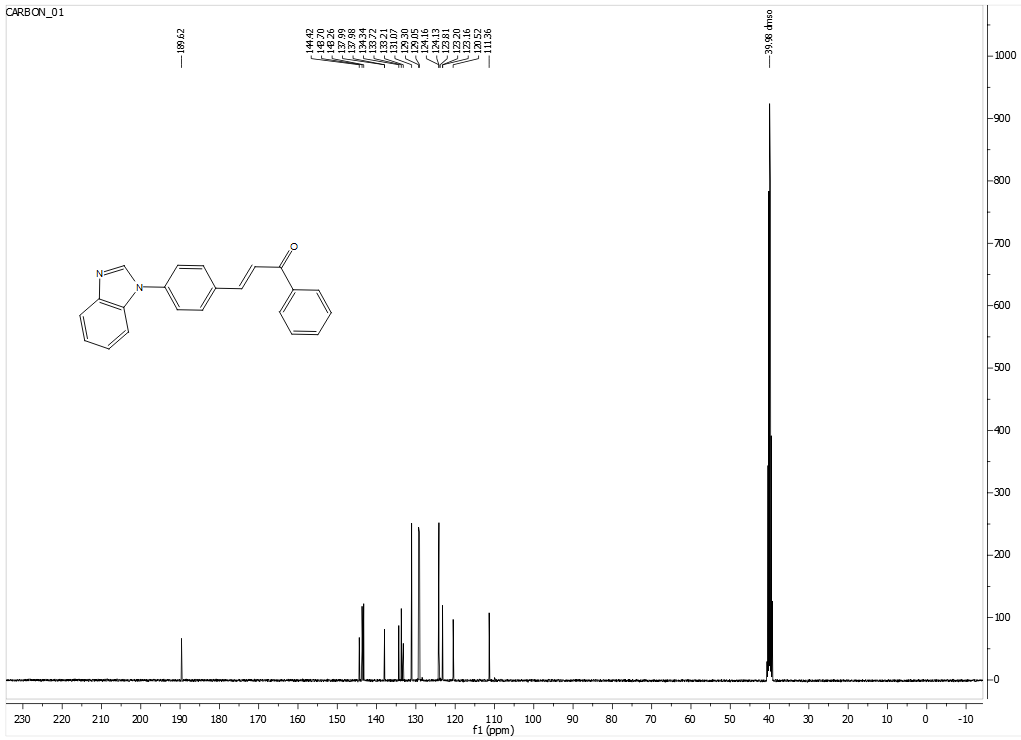
**Fig. S2.** Compound 1 ^13^C-NMR spectra


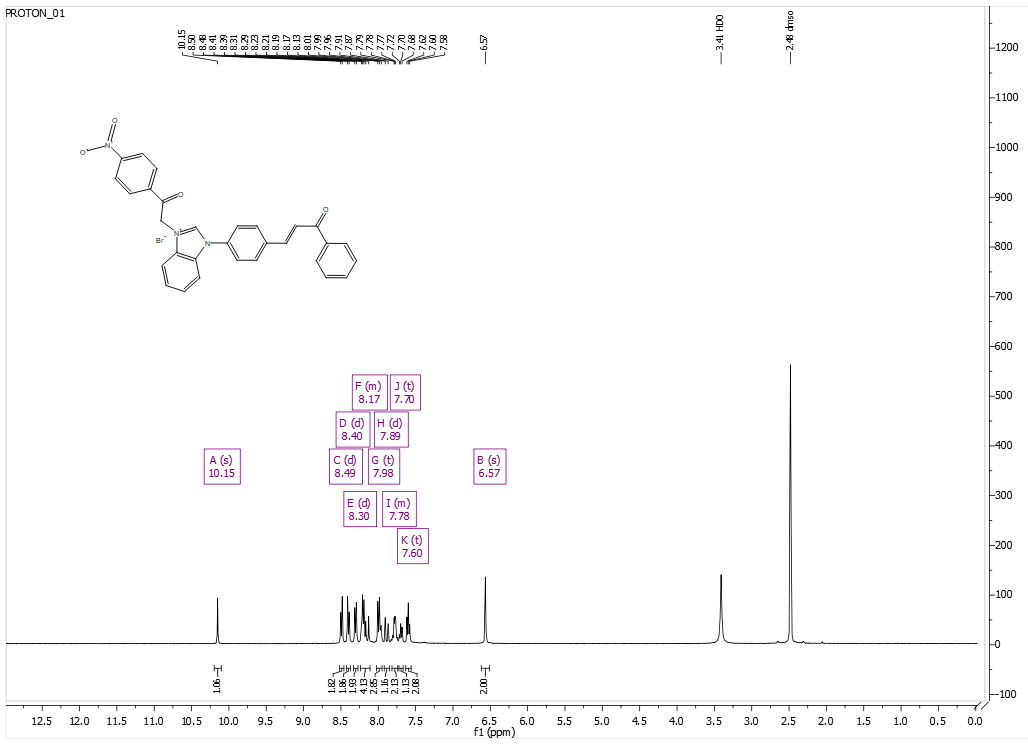
**Fig. S3.**  Compound C1 ^1^H-NMR spectra


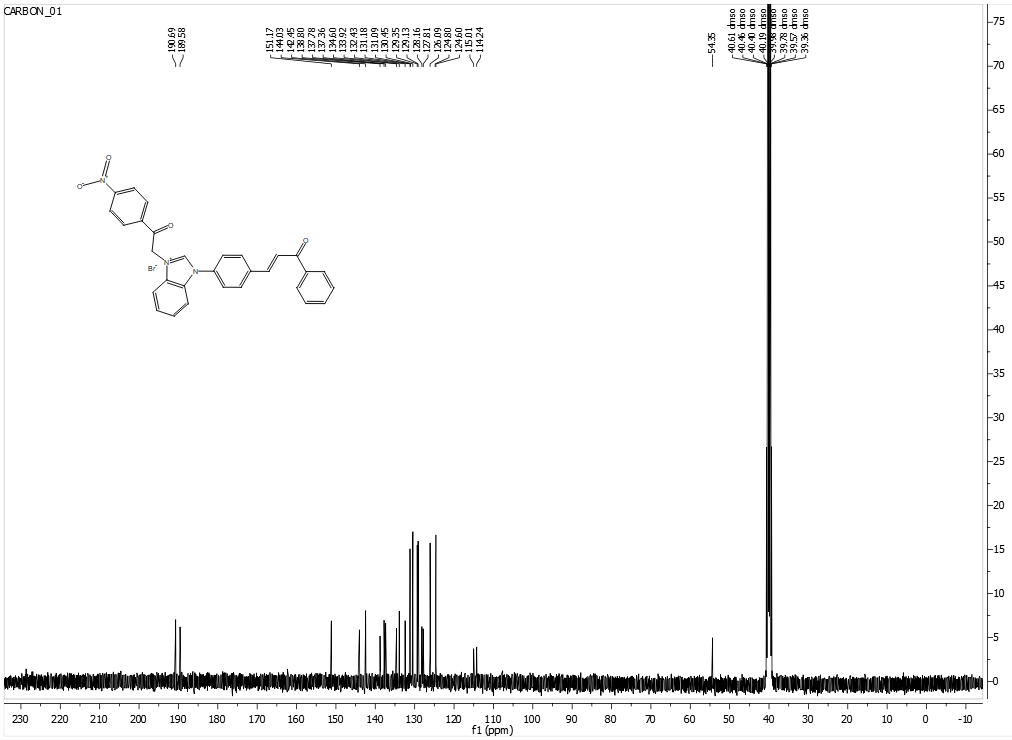
**Fig. S4.** Compound C1 ^13^C-NMR spectra


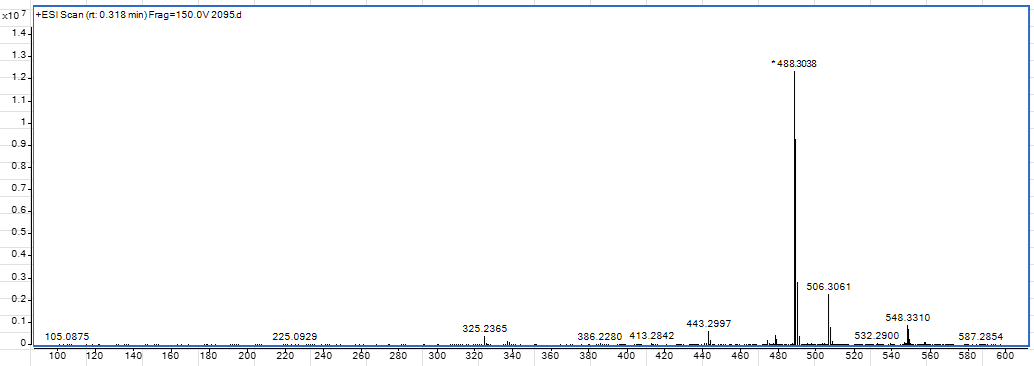
**Fig. S5.** HR-MS spectrum of compound C1


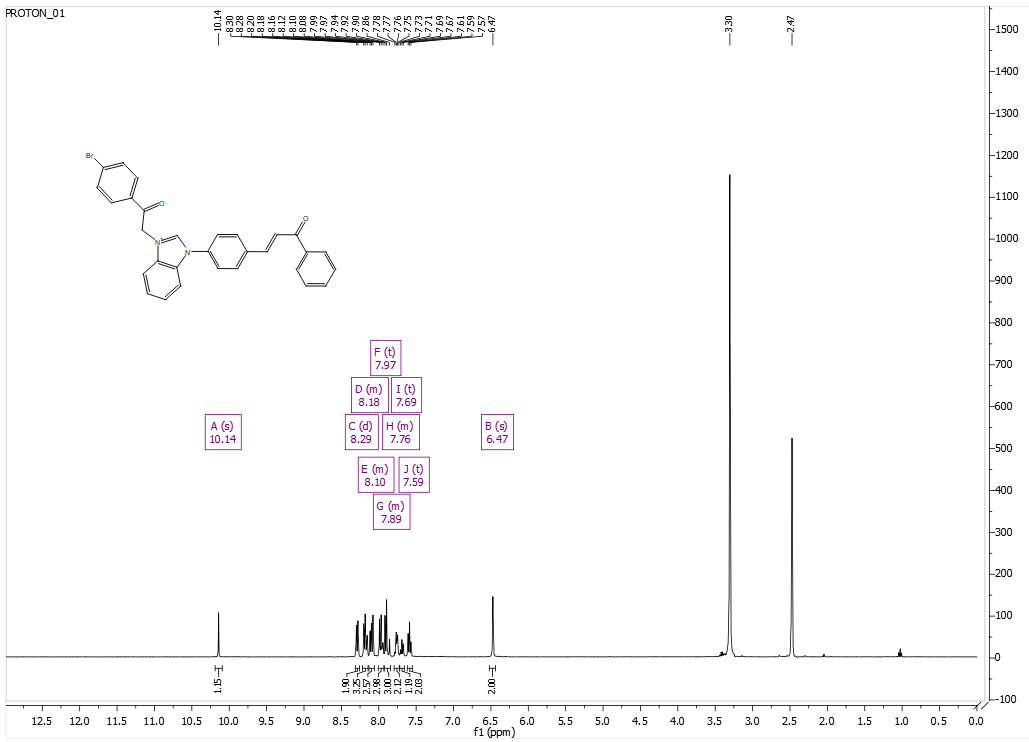
**Fig. S6.** Compound C2 ^1^H-NMR spectra


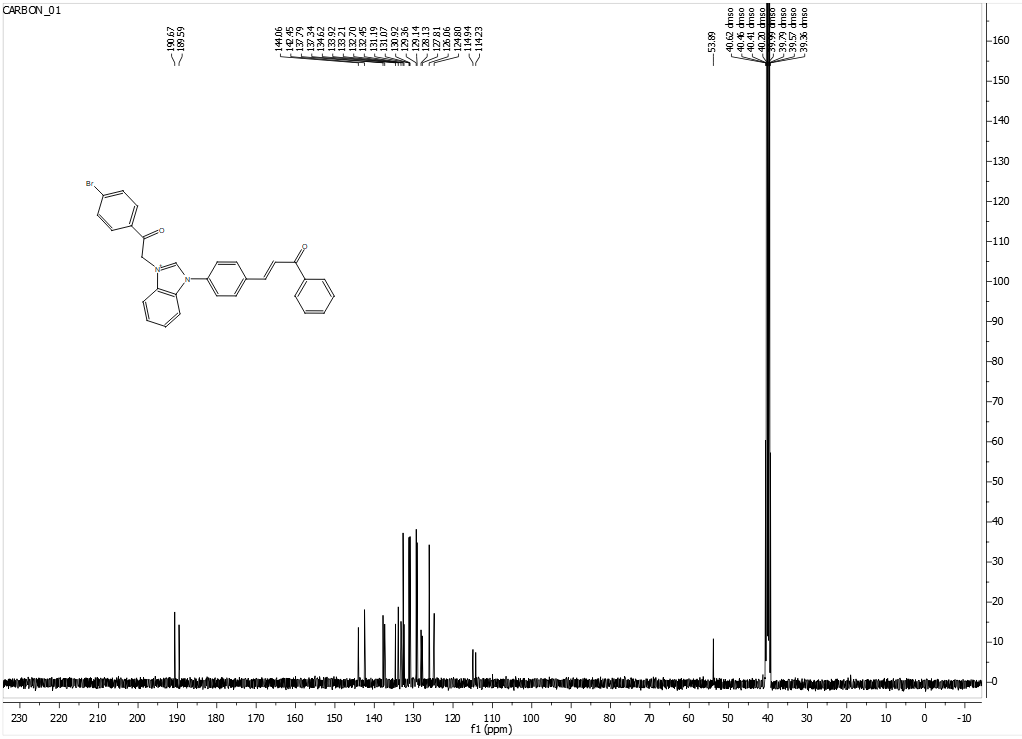
**Fig. S7.** Compound C2 ^13^C-NMR spectra


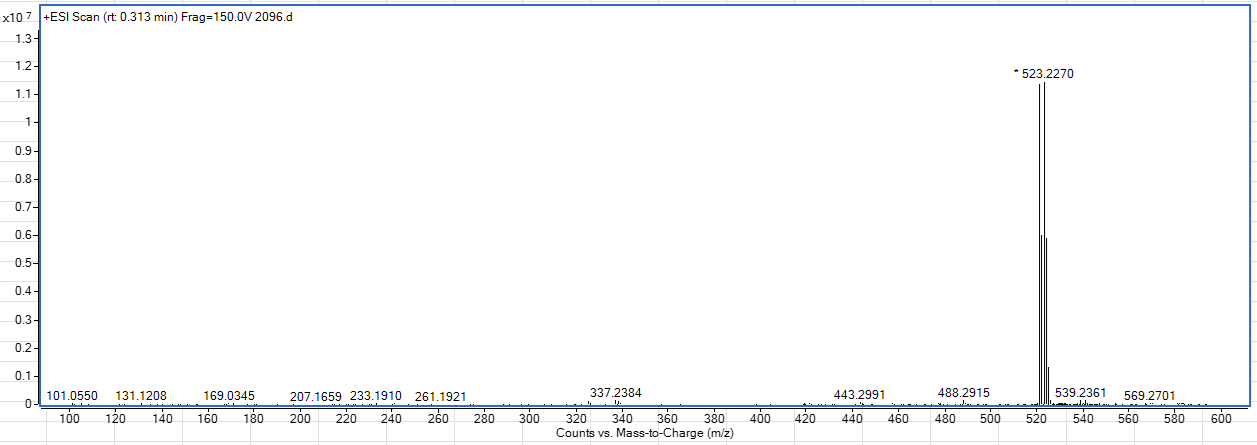
**Fig. S8.** HR-MS spectrum of compound **C2**


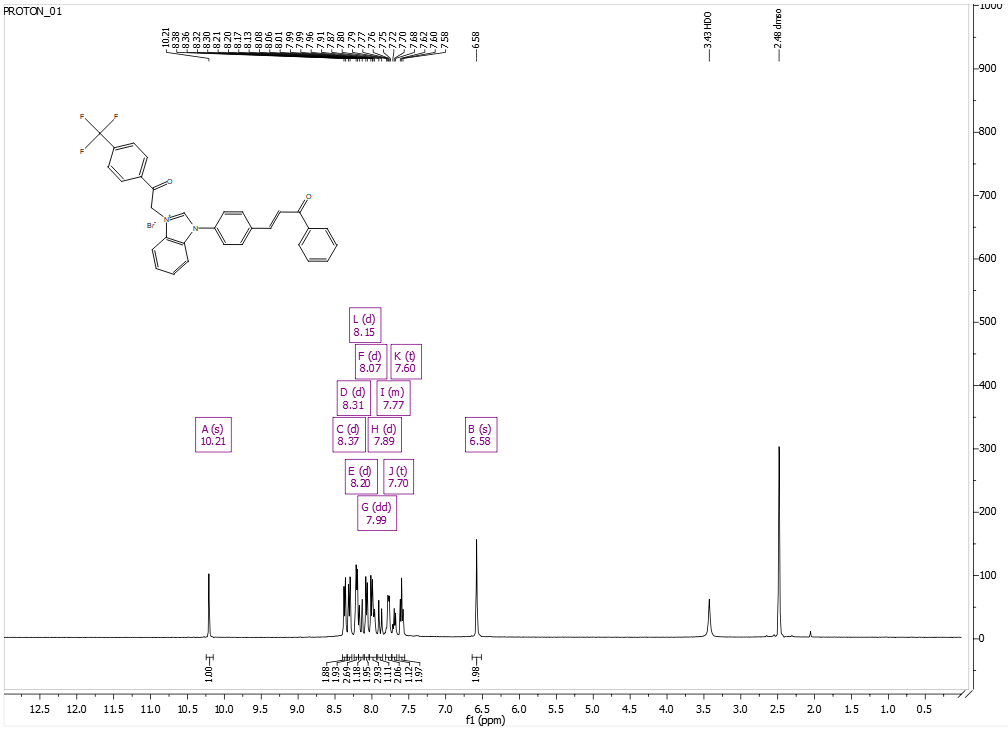
 **Fig. S9.** Compound C3 ^1^H-NMR spectra


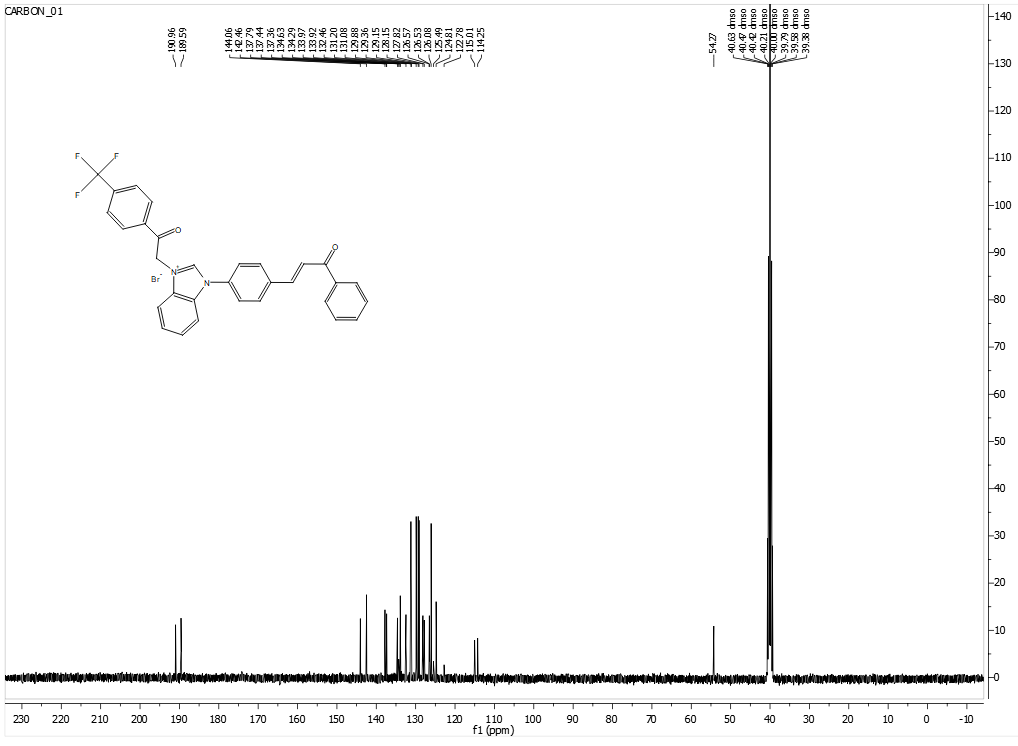
**Fig. S10.** Compound C3 ^13^C-NMR spectra


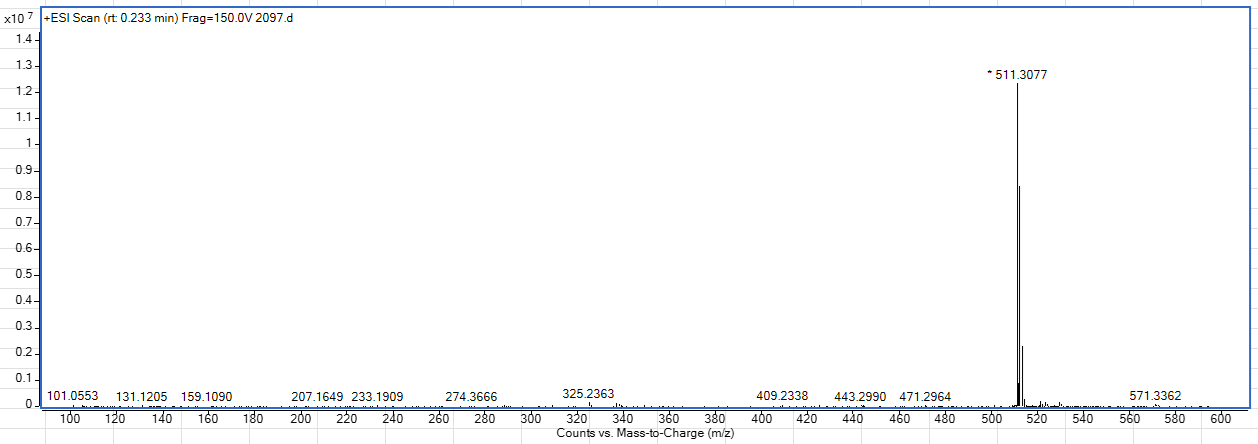
**Fig. S11.** HR-MS spectrum of compound C3


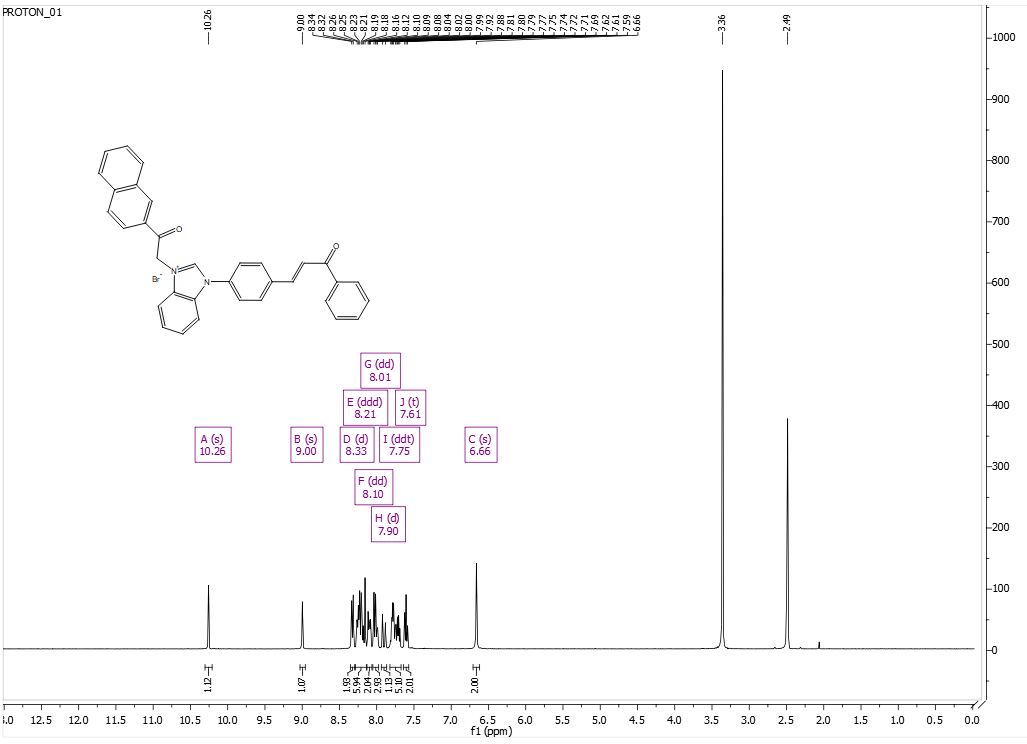
**Fig. S12.** Compound C4 ^1^H-NMR spectra


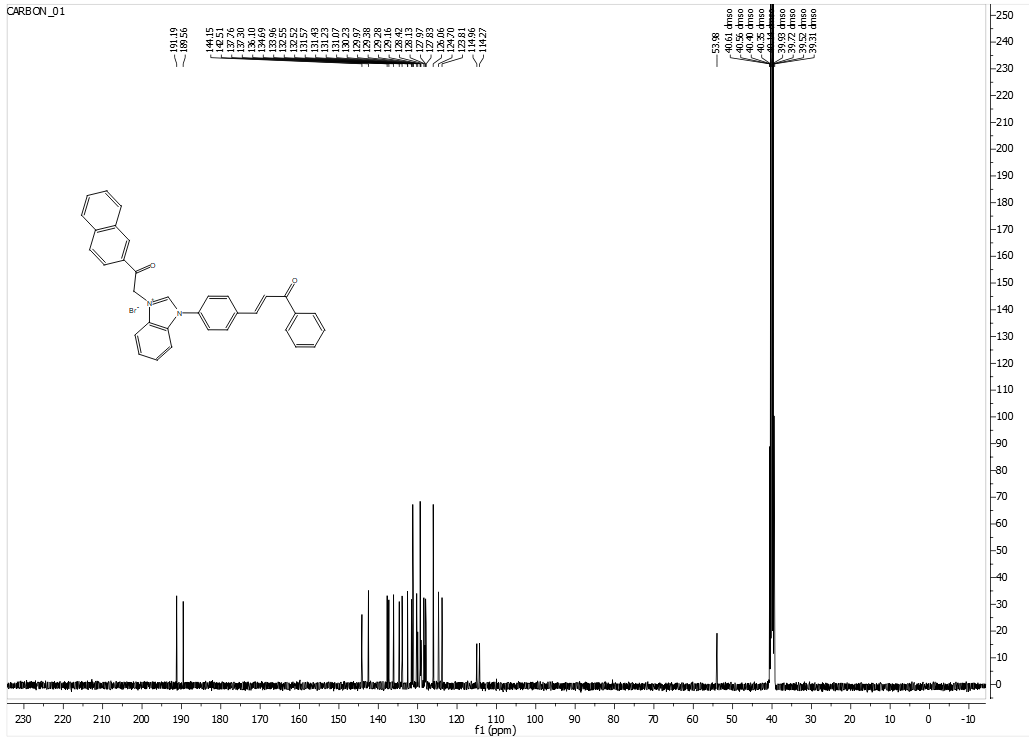
**Fig. S13.** Compound C4 ^13^C-NMR spectra


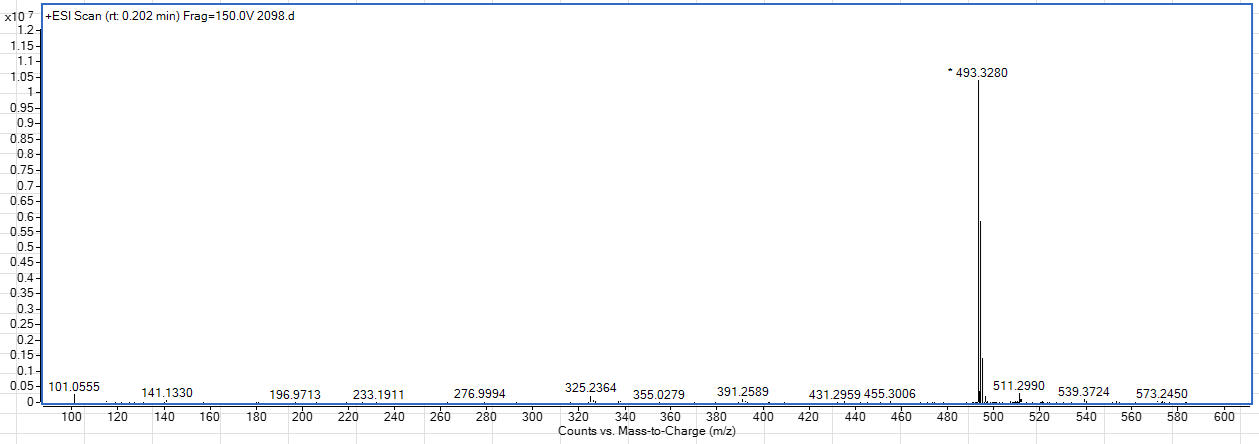
**Fig. S14.** HR-MS spectrum of compound C4
